# Clinical Collections and Observations in Surgery

**Published:** 1847-01-01

**Authors:** 


### Clinical Collections and Observations in Surgery, made during an attendance on the Surgical Practice of St. Bartholomew's Hospital. By *W. P. Ormerod.* 8vo. pp. 300. Longman, 1846.

All who are acquainted with the Metropolitan Hospitals must be aware of the exemplary manner, as a general rule, in which the medical officers perform the immediate duties devolving upon them. Let but the sufferings of the most wretched outcast be sufficiently great to secure his admission within the walls of these noble institutions, and he has skill and care lavished upon him equal to that which the wealthiest noble could command: with, too, the additional advantage of the treatment of his case being conducted in the presence of the most competent and most formidable of critical tribunals—that formed by aspiring colleagues and senior students. So, too, the business of hospital teaching, with some exceptions, is far more satisfactorily conducted now than heretofore; and it is the student's own fault if he do not avail himself of the invaluable and never to recur opportunities of improvement which are now so freely opened up to him. But with all this we think the Hospital Medical Officer is seldom sufficiently impressed with the duty which devolves upon him, as respects the profession at large, of freely communicating the results which his position has enabled him to achieve. We hold that no man is morally justified in taking any such important office who is forgetful or careless of this obligation, inasmuch as he prevents another conferring an important benefit upon mankind, which he himself is either incompetent or unwilling to accomplish. The Guy's Hospital Reports furnish an excellent example of what may be done by the united efforts of men who are alive to the duties of the offices they hold; and the names of Brodie, Latham, Ashwell, &c., point to recent admirable individual exceptions to the general apathy we are deploring. May the surgeons of St. Bartholomew's arouse themselves, and follow so excellent a lead! In the mean time we have Mr. Ormerod, a most industrious student, and late house-surgeon of that hospital, coming forward with his "Clinical Illustrations," the fruit of nine years assiduous observation in the surgical wards. It is just such a work a hard student ought to produce, and is not liable to an objection that attaches to the published lucubrations of several junior practitioners. If many of the senior members of our profession have manifested an undue remissness in wielding the pen and delivering opinions which would be listened to with attention and instruction, no such coyness has been exhibited on the part of some of the younger portions of the fraternity. With many of these, a few years' hospital practice (especially if a portion have been passed in a foreign country), seems to confer the right of discussing all matters *ex cathedra*: and, in elaborate original articles in the periodicals, or even substantive treatises, subjects are disposed of with readiness and precision, upon which the sages of our profession see cause yet to hesitate and doubt. Mr. Ormerod confines himself to a concise record of the most interesting facts which have come under his notice, adding here and there general observations legitimately derived from the extensive field of observation that has been laid open to him. His work is divided into twenty chapters, treating successively of the more important surgical diseases, the last six being devoted to a consideration of the relative merits of mercury and iodine in the treatment of syphilis. This essay obtained for its author the award of the Jacksonian Prize in 1842.

The miscellaneous character and conciseness of the descriptions of the first portions of the work render any continuous analysis of these out of the question. We shall therefore content ourselves with reference to some few topics, noticing the chapters on the treatment of syphilis somewhat more at length.

*Fracture of the Thigh.*—Some of the signs of *fracture of the neck of the femur* are frequently absent, although it is rare to find them all so. However doubtful they may be, all injuries of the hip occurring in old people must be narrowly watched, as several days may elapse before it can be positively declared that a fracture has not taken place. "The result alone can test the point, and nearly always the result is, that the limb is broken." A woman, æt. 66, who had been knocked down on her right hip, was admitted. Shortening of about an inch, but without the slightest *eversion,* was observed. The absence of this was explained after death by the partial laceration of the capsule and the irregularity of the fracture, the lower fragment being wedged into and overlapped by the upper.*

In *treating fracture of the thigh,*

"The bed of Mr. Earle, and the bent position on the side, have been discontinued latterly in great part at St. Bartholomew's, for the long straight splint. The limbs unite better, the trouble is less, and the expense is much less; the high beds being very dear, and spoiling a blanket each time that they are covered. There is also another evil in hospitals; if fleas and bugs once get into a high bed, it is very hard to get rid of them. There are, however, some cases in which a high bed is good. If a patient has two broken thighs or legs, a high bed allows the chest to be raised, and thus he can move and is less liable to risk in vomiting. It is said, that a fracture in the upper third is not so liable to rise on the high bed, as the lower portion can be brought to meet it. This is very doubtful, indeed, in practice. Thighs broken in the middle, and not fully extended, generally unite with the lower end of the upper part on the outside, or in front of the upper end of the lower portion. This is very hard to prevent on the high bed, and a mere matter of chance on the side; but with a long splint, and a long inguinal band right up to the axilla, as high as can well be done, it is partially prevented." P. 45.

*Fracture of the Leg.*—A case is cited in which a man walked from Highgate to Smithfield (4 miles) in four hours, the tibia being broken across its middle, and the fibula somewhat lower down!

When fracture of the *fibula* is difficult of detection, Mr. Ormerod recommends the following procedure:—

"Place the right hand with the ends of the fingers on the fibula midway between its two extremities, and press it towards the tibia. Even in the stout

* For a few interesting observations on the diagnosis of this affection, by M. Velpeau, the reader is referred to the Foreign Periscope of our present number.

fibula of a healthy man, the bone will often play between its two extremities under this pressure. If a general easy movement is found, by passing the left hand up and down, to take place all the way between the two attachments of the fibula, fracture is very improbable indeed, whilst occasionally the pain and amount of free motion produced by this pressure shew that fracture has taken place. When the tibia and fibula are broken near the ankle-joint, without any bruise or displacement, the detection of crepitus is sometimes very difficult, and can only be ascertained by firmly grasping the ankle with one hand, and the lower-third of the leg with the other, and attempting to move the parts slightly on each other. Unless the greatest caution be observed, fracture in this situation may pass unnoticed. Occasionally any displacement is so entirely wanting, that it is necessary to hold the part just above the injury firmly with one hand, and to press the lower part firmly backwards with the thumb and fingers, when the acute pain with a very slight crepitus shew the existence of fracture." P. 51.

Mr. Ormerod thus describes the plan adopted in Mr. Lawrence's wards for preventing the bones from projecting unduly forward after fracture of the leg, situated low down.

"The leg is placed in the bent position on the outside, with a common side splint placed above and below, slightly hollowed out to fit the leg. In addition to these, two straight splints are used, padded on one side, one of sufficient length to extend from the patella to the upper part of the lower-third of the leg, the other long enough to reach from the hollow of the knee to beyond the heel. If the straps be now passed round the leg, including the shorter of the two straight splints on the front, and the longer splint on the back of the leg, along with the two hollow splints on the upper and under side, the tibia and fibula above the fracture will be pushed backwards, whilst the foot with the part below the fracture is pressed forwards. In this manner the tendency of the tibia to pass forwards, after simple dislocation or fracture near the ankle, is effectually prevented." P. 52.

*Dislocation of the Thumb.*—The difficulty which sometimes occurs in effecting the reduction of this, must be known to most of our readers. Mr. Ormerod states that the suggestion offered by Mr. Wormald, some years since, of making the extension with the elbow and wrist, as well as the metacarpus and joints of the thumb, considerably bent, has been put into force at St. Bartholomew's with the best results. Not only is the reduction more easily operated on than when the parts are kept extended, but cases have been in this way sometimes reduced which were otherwise irreducible. The patient should be seated on a stool with his back to the surgeon, and the bent arm and hand brought over the shoulder and behind the neck while extension is made. Other cases, however, resist all means whatever. The difficulty in some of these seems to have been produced by the interposition of the tendon of the flexor longus pollicis. To illustrate this point, Mr. Stanley performed the following experiment on the dead body.

"An incision was made across the distal joint of the thumb, resembling that occurring in compound dislocation. The sheaths of the tendon and lateral ligaments were divided, whilst the tendon of the flexor longus was allowed to pass behind the middle phalanx, and consequently between the two bones at the joint, which was dislocated. Great difficulty was now found to exist in reducing the dislocation, which was found to increase in proportion as the sheath of the tendon was divided higher up, and the tendon allowed to pass behind the phalanx higher up, and consequently more directly through the middle of the joint. When, by lateral twisting, the tendon could be brought round the extremity of the bone to its natural situation, the part was immediately reduced; so difficult however was the reduction, that it was only by three persons trying with all their force, one after another, that the thumb of a dead man, artificially dislocated, could be reduced." P. 67.

*Discharge of Serous Fluid from the Ear in Fracture of the Skull.*—Our readers are aware (Med. Chir. Rev. N.S. VI. p. 547) that this subject has of late excited some attention and speculation in the French medical journals. The cases therein related were uniformly fatal; but Mr. Ormerod alludes to examples in which the patients sufficiently recovered to leave the hospital. In 13 cases of injury of the head either blood or this serous fluid flowed from the ear; and, of these, seven recovered and six died. Of the seven, two suffered from the watery discharge and five from bleeding. Of the six fatal cases, two had bloody and four watery discharge. As in the French cases, the petrous portion of the temporal bone was always found broken on the side upon which the discharge occurred: but it is not stated whether in the instances of serous discharge the fracture was in the form of a mere fissure, through which M. Laugier supposes the watery portions of the blood to filter—a very lame explanation it must be admitted.

*Treatment of Enlarged Subcutaneous Bursæ.*—"When matter has formed in them, the only means is the evacuation of the fluid by a free opening: this is unattended with danger, and followed by a rapid and complete cure. When, however, the bursa is recent, the skin thin, and the fluid probably a mere increase of the natural secretion of the cavity, the employment of blisters, or the external application of the tincture of iodine, is the best means of lessening the swelling; but it will probably return. For a complete cure, or in those cases where the swelling does not yield to the application of blisters, or to the external application of iodine, more especially if the swelling be not large, the best plan of treatment is to introduce a fine thread through the swelling, and use it as a seton. On the second day this thread generally causes considerable pain, and requires withdrawal. A small quantity of puriform fluid passes for a few days through the opening, after which the swelling gets gradually less, and contracting, is completely cured. Very frequently the bursa suppurates so freely as to require a free opening, the hole for the thread having closed. Although this is an extra source of pain, yet the cure is more complete, and quite compensates for this accident. Removal of bursæ simply for their inconvenience is a serious matter." P. 90.

In his Chapter on *circumstances influencing the convalescence of patients*, Mr. Ormerod enters into an examination of some of the causes of death in the surgical wards. Prominent among these stands the *prior intemperate life of the patients*. Some have been great beer, and others spirit drinkers; and both classes, although in different modes, find themselves ill able to contend with casual injuries however slight, although, prior to the occurrence of these, they might have deemed themselves in good health; and indeed some of the large beer drinkers, as draymen, would be considered by most persons as very robust. Although the *granular liver* has attracted most attention as the disease of drinkers, M. Ormerod states, the two organic affections which are most to be dreaded in the intemperate patient of the surgical wards are the *granular kidney*, and slight but general *em-*

*physema pulmonum,* with a *dilated, but not always much diseased heart.* "The kidney and the lung in these cases are not generally in an extreme state of disease, but disease in a slight degree is very generally distributed: thus there are no bladders or extreme emphysema of any one part of the lung, but the cells in every part are more or less affected, whilst the kidney is not contracted, but mottled and dotted in every part." The low fever, again, which so frequently prevails in the metropolis, is another cause of the aggravation of slight affections into serious or fatal maladies. Under its influence a mere gonorrhœal excoriation may be converted into sloughing phagedæna. In surgical diseases themselves, whether of a serious kind, such as compound fracture, or of a more trivial description as in simple injuries, the concomitant febrile action may take on all the characters of low fever, rendering the administration of support as necessary, but less useful, as in simple low fever. The post-mortem changes observed are also alike in the two diseases, except that, in the surgical disease, slight but extensively diffused organic changes are found, as well as the more transitory ones induced by fever.

"The three chief affections destroying patients after operations and injuries—the general habit produced by drinking; secondly, organic disease in the lungs and kidneys, especially emphysema in the former, and granular disease in the latter; and thirdly, tubercle—act very differently, and at different periods. During the early period, and often for weeks after operations, patients labouring under tubercular disease do well; and it is often only at the absolute return to health, rather than during the recovery from the operation itself, that the effects of tubercle begin to shew themselves. Organic disease produced by drunkenness, and habitual drunkenness, act differently: the organic disease presses heavily at every period, and may destroy life either early or late; but the mere habits of the drunkard shew themselves chiefly at a very early period. The patient, who nearly sinks from his unsound organs within the first few days, often lags on for days and weeks in danger; but the man who has simple delirium tremens is taken ill directly, and often dies, but if he recovers from his delirium, he generally gets well from the operation, and sometimes quickly." P. 101.

*Amputation.*—Mr. Ormerod thus states some of the reasons in favour of the flap-operation, which seems at last to be displacing the circular so long adhered to (why it would be difficult to explain) at St. Bartholomews'.

"In the thigh and leg, after amputation, it not uncommonly happens that everything looks well for a few days, but that then some matter forms, or the limb jerks, or is hot, or the skin gets just a little tight at one part over the bone. In these cases the flap-operation succeeds better than the circular, for it rarely happens that the skin of the circular operation can be got well forward again after it has once begun to retract, or become tight, whilst the mass of muscle and soft parts of a flap can often be brought down again after they have retracted very considerably. In the thigh, puncture of the artery, above its division, is readily avoided in the flap-operation, and cannot well be done in the circular. In the leg, the artery may readily be punctured in passing the knife behind the limb, and wounded above its division; still this is no real objection to the flap-operation below the knee, as the same accident may happen from the use of the catlin. The rapidity of the flap-operation, as compared with the circular, is some advantage, but the whole operation is not necessarily shorter, for the number of arteries to be tied in the former case is generally greater than in the latter. During the last few years the double-flap operation has been performed upon a large number of patients at St. Bartholomew's by Mr. Stanley, and with the best result. In many of these cases, at their termination, the full soft condition of the face of the stump, the complete depression of the bone in the line of the union of the flaps, or beneath the front flap of the thigh, have been most marked, whilst the effects of inflammation, in rendering the stump tense, have been very much less than where the same accidents occurred after a circular operation." P. 131.

*Hæmatocele* is very rarely found occurring *spontaneously.* We have cited some cases of this in our April number (p. 661), and Mr. Ormerod furnishes us with another. A man, æt. 71, was admitted with a large, red, fluctuating tumour on the right side of the scrotum. Three weeks before there was nothing remarkable in the part, when, after going to stool, he perceived a swelling which appeared to have arisen spontaneously and was attended with no pain. It rapidly increased in size for two days, and afterwards more gradually. Thirty ounces of a bloody fluid were evacuated by a puncture, and when the man left the hospital, about six weeks afterwards, the testicle was of its natural size.

The contents of a hæmatocele may vary much in consistency, but it does not seem that this circumstance influences the rapidity of their absorption. Sometimes this takes place very rapidly. A man was tapped for a hydrocele, which then became converted into a hæmatocele. The blood was evacuated by a trocar in nine days, but the tumour was reproduced. Cold lotions were now applied, and in three weeks the scrotum was nearly its natural size.

The cutaneous veins of the scrotum are not in many cases the source of the bleeding; for, although this may be sufficiently abundant to fill a large hydrocele in a few hours, there is little or no ecchymosis of the scrotum. These veins, moreover, are of but small size, so that, even in the case of inflamed scrotum, it is sometimes difficult to obtain blood from them. Again, supposing they furnished a sufficient supply of blood, would this find its way through the opening left by the trocar into the tunica vaginalis, rather than into the cellular tissue? Judging from what takes place when the canula slips from the aperture into the tunica during injection, we cannot suppose so.

"Where does the blood really flow from in these cases? It flows rapidly, without ecchymosis of the scrotum, and often flows again and again, if it is repeatedly evacuated by the trocar. Three sets of vessels may pour out blood under these circumstances. The vascular lining of the tunica vaginalis has appeared on dissection to have been the source of the effused blood (Ed. Med. and Surg. Jour., v. 38, p. 325), in addition to which the plexus of veins on the cord might readily pour out blood, as in the case of spontaneous hematocele already related. In addition to these vessels inside the sac, there is another set of vessels distant from the skin, large in size, close on the tunica vaginalis, quite out of sight, and more or less closely connected with the spermatic plexus of veins, which is very sparingly supplied with valves. If, in the dead body of a person labouring under hydrocele, an incision be made over the ring, and the hydrocele be pulled out of the scrotum, the cutaneons veins and skin are left, and the cord with the hydrocele is lifted up. But the hydrocele is not bare, but running on its surface, and almost in its coats, may often be seen veins of considerable size, not passing to the perineal veins and the cutaneous veins of the thigh, but connected more or less with the large veins of the cord itself. Here is a possible source of blood, which the eye would not detect, and which might pour out blood under those very circumstances, which are not clearly explained, if the blood be supposed to come from the cutaneous vessels of the scrotum alone." P. 162.

*The Administration of Food by Enemata.*—Perhaps this mode of conveying nutrition into the system in cases in which the patient either cannot or will not swallow, is not sufficiently resorted to, although, truth to say, the examples of its efficacy, in cases in which it has been employed, are not sufficiently numerous to afford much encouragement. Mr. Ormerod, however, furnishes a well-marked one. "A man of about 20 years of age was admitted with his pharynx opened and the glottis exposed. He was unable to articulate, and vomited frequently through the wound, for an hour and a half, fluid mixed with blood. On the 2nd day he had an enema of milk. From the 2nd to the 41st day he took daily, in three enemata collectively, two pints of broth made from rather more than one pound of beef. His hunger was always appeased by the enemata. When his bowels were confined some salt was added, which was sufficient to open them. Once some wine was added to the injection. On the 41st day the injections were omitted, and food was given by the bowels (?) On the 51st day the wound was nearly healed, and the man looked well, and in tolerably good condition. He could however only speak in a whisper."

### Treatment of Syphilis.

Nearly one-third of Mr. Ormerod's work is occupied with the Essay on the Comparative Merits of Mercury and Iodine in the Treatment of Syphilis, which obtained for him the Jacksonian prize. It is a valuable record of the practice of St. Bartholomew's, where a large number of beds, considerably more than a hundred, we believe, are devoted to the reception of venereal patients. We are glad to find that the surgeons of that hospital still hold fast to their faith in mercury, convinced as we are that a more mischievous practice does not exist than that of attempting to treat the disease without it. Mr. Ormerod seems to us to have estimated the value of the two drugs very fairly, and to have well deserved the award that was made him. He properly insists, in his first chapter, upon the entire removal of the *induration* before the cure can be considered complete; but we have not been in the habit of attaching so much importance to the *local* use of mercury as he does.

"When the ulcer is running its usual course, is still unhealthy on its surface, and presents all the signs of progressive disease, the local application of mercury in certain forms is generally attended with the best results. So frequently, and with such decided benefit, has it been applied while the sore was still spreading, that it has appeared to be one of the most powerful means in correcting the unhealthy condition of the primary venereal ulcer with induration, and converting it into a healthy granulating sore; and, notwithstanding the descriptions of the bad effects of local mercurial applications to ulcerating sores, opportunities occur, from time to time, in which one may observe the greatest benefit not unfrequently following their application. * * * * * * * * * * * The application of mercury in a fluid form to indurated ulcerating sores, appears to act more beneficially than when mercury is employed in the form of ointment. It is applied more equally and easily, and only needs occasional renewing. The black and yellow washes are both thus employed: whilst, however, the latter is generally applied to ulcerations of the integuments in secondary syphilitic affections, the former is generally applied to primary ulcerations of the genital organs and parts round. For these reasons the application of mercury to the primary sore, in the ulcerated stage, is best performed by employing that substance in solution, as in that form it comes into accurate contact with the diseased surface. When, however, the ulceration has healed, the employment of mercurial lotions is not attended with such decided benefit, on account of the entire condition of the skin over the indurated part. * * * * * * To indurations on the mucous membranes or integuments of the genital organs, after all important inflammation has subsided, the strong mercurial ointment may be applied with the greatest advantage. If a portion of the ointment be occasionally rubbed on the hard part, and a piece of leather covered with it be constantly applied, a decided diminution is sometimes observed to occur in the size of the part." P. 214.

Because a case is of old-standing and obstinate character we are not to have recourse to violent courses of mercury; the medicine must be then *mildly administered for a very long period.* Some of the best examples Mr. Ormerod has seen of its successful application have been in certain cases under Mr. Lawrence's care, in which small doses of *hydrarg. cum cretâ* were continued perseveringly. When the quick and decided influence of mercury is required, or its effects on the bowels feared, *frictions* form the best means of employing it; but, in the majority of cases, the convenience of the *blue-pill* will secure it the preference. In the *phagedœnic* form of ulcer the mercurial action should be rapidly induced by calomel and opium, as recommended by Mr. Lawrence. Some of these cases in the wards of that gentleman have improved as rapidly after the induction of mercurial action as cases of syphilitic iritis. After the phagedænic action is checked the cure may be completed by a milder preparation; and in these cases, in bad and unhealthy subjects, where the sore after a while becomes stationary, the hydriodate of potash with sarsaparilla and a good diet may be advantageously substituted, the patient being removed also into a purer ward. Certain phagedænic sores, attended with and maintained by a high degree of surrounding inflammation, require to be treated with local depletion and purgatives; while in other feeble, haggard patients, presenting instances of nervous rather than vascular disturbance, wine and opium are indicated. So successful has mercury been found in treating the various primary sores at St. Bartholomew's that iodine has been but little used, and apparently with no great advantage. It seems, however, to aid, when added to sarsaparilla, in recruiting the exhausted frames of certain patients in whom the disease has long lingered and become combined with secondary symptoms.

Among the great number of *secondary venereal affections* seen at St. Bartholomew's, those of the *skin* predominate. The papular and scaly eruptions, with an irregular mottling of the skin, are the most common; next the tubercular and pustular; and lastly, the phagedænic pustular disease termed rupia. Vesicular eruption is seldom if ever seen. The *papular* eruption in its earlier stages requires antiphlogistic proceedings, after which, mild mercurials, to the extent of just touching the gums, form the most efficacious treatment. The warm-bath and in some cases good diet and tonics are valuable adjuvants. Iodine is certainly less efficacious, and in obstinate cases very much less. In no form of disease is medicine so markedly useful as mercury in *scaly* syphilitic eruption. So useful is it that iodine has rarely been tried. This, however, possesses considerable efficacy when the affection is but slight. Syphilitic *tubercular* eruption, when the patient's health is good, will also yield to a mild mercurial course; but in the mildest form, and when the patient's general condition is unsatisfactory, iodine is preferable. Mercury too, is adapted for the *pustular* form, when the patient is not feeble, and there is no disposition of the pustules to degenerate into ulcers; but it requires careful watching. The *secondary ulcers of the skin*, which are met with chiefly in the most wretched class of patients, who have employed neither care or cleanliness, and whose general health is more or less ruined, have been attributed by many to the employment of mercury. This, Mr. Ormerod considers an erroneous view, believing that a cautiously conducted course of mercury has no tendency to produce them, admitting, however, at the same time, that mercury given rashly or at random, and the patient exposed the while to every injurious physical influence, may induce their formation. They usually present themselves under three different forms. They may be seen (1) as numerous small round ulcers scattered over various parts of the body, being usually accompanied by other secondary symptoms. The patient's health is not generally sufficiently bad to prevent the cautious administration of mercury, which, with the local use of the yellow wash, will usually effect a cure. 2. Large, semicircular, phagedænic, shallow ulcers form the symptom for which iodine may be most advantageously given. 3. Round, excavated ulcers, occurring in small numbers, or singly, attended with great pain, and sometimes extending very deeply. Opium, nutritious diet, and iodine are here indicated, the blackwash often forming a suitable local application. In the different ulcers mentioned, various measures one after another are tried in vain in some cases, and in such, the corrosive sublimate gr. $\frac{1}{12}$ ter, continued for a long period, sometimes succeeds where everything else has failed.

We must pass Mr. Ormerod's account of these medicines in the other secondary symptoms more rapidly under review. *Ulceration of the throat* is upon the whole better treated by mild mercurials than by any other measure. In the *excavated ulcer* this is especially the case, if cinnabar fumigation be simultaneously used. Iodine neither cures so rapidly or so completely as mercury. In a class of cases which combine the appearances of excavated ulcer of the fauces and sloughy ulceration of the pharynx, the local use of cinnabar, and the internal employment of iodine form a very successful mode of treatment. Persons labouring under sloughy or foul ulcers generally require good diet and wine or porter, and, in such, iodine is of the greatest service. Since iodine has been employed in these cases, fumigations have been much discontinued. Where, however, the object is to clean the sore very rapidly, other means having failed, and the patient is strong, fumigation will often be still found a valuable means; although its administration requires care in consequence of the ease with which a profuse and injurious salivation may be induced by the application of the cinnabar to a large sore. In *ulceration of the larynx,* iodine, conjoined or not with mercurial fumigation, is a most valuable medicine. The patches of *ulceration* seen on the *tongue* are relieved by the mercury which is indicated for the fissures of the lips and scaly disease of the skin which usually accompany them. In a form of ulceration of the edge of the tongue and corresponding portion of the cheek, exactly resembling that induced by salivation, iodine effects a rapid cure. Excavated ulcer of the upper surface of this organ is occasionally seen, and a slight mercurial course is the appropriate remedy. The *fissures* occurring at the edge of the tongue often heal readily to recur again and again. They must be treated by iodine or mercury, according to the accompanying symptoms, and if these do not exist, by a mild course of mercury.

*Hernia Humoralis* is usually of simple treatment. After the acute stage has been checked by antiphlogistics in a day or two, the *hydrarg. cum cretâ* 2½ gr. ter, continued steadily, has been found of great benefit at St. Bartholomew's. The syphilitic testis generally yields very readily to mercury; but in some cases it may require careful persistence in its general and local use. Messrs. Lawrence and Stanley have found the application of the strong mercurial ointment or liniment to the scrotum, a very beneficial measure.

In respect to affections of the *bones and periosteum,* in no form of disease is iodine of greater use than in the dull aching *pains* of the long bones. It mostly relieves immediately or speedily, but if it does not do so shortly much benefit does not usually accrue from its prolonged employment. Since the introduction of this medicine, affections of the bones and periosteum have so frequently been cured, that incisions are now seldom called for. Cases, however, occasionally do occur in which these are advisable, others in which mercury is more useful than iodine, and others again in which neither medicine gives any relief. In *nodes,* unaccompanied by acute inflammation, iodine is a most excellent remedy. When the node is soft, mercurial ointment on a piece of soft leather, firmly applied over it, materially diminishes the swelling, and does not cause irritation or ulceration even if the skin be very thin. Sometimes this is so thin as to seem on the point of bursting; but we must cautiously avoid opening it in consequence of the troublesome ulcer which may result. Fluid and recent nodes admit of complete cure, while old bony ones are rarely if ever removed, although the pains accompanying them may be relieved. Iodine has been found very useful in *gonorrhœal rheumatism,* given after the constitutional irritation has subsided, or at once if this is not considerable. The cure, however, is not complete, the patient being long tormented by occasional pains, and it seems gradually only to wear itself out.

Cases of *syphilitic ulceration of the eyelids,* described by Mr. Lawrence in his treatise, are not so effectually treated by iodine as by a mild but effectual course of mercury. Sometimes the papular eruption of the skin extends to the *conjunctiva,* producing a very troublesome affection. The application of a few leeches to the lower lid much relieve the accompanying irritation. Iodine and mercury exert no influence apart from their action on the eruption of the skin. Although these cases usually do well, they are occasionally tedious and difficult of cure, the eyes being long left in a very painful and irritable condition. Benefit is sometimes derived from rubbing tartar-emetic ointment into the nape of the neck. Upon the vast utility of mercury in *Iritis,* we need not detain our readers, merely stating that in the *chronic form* of the disease Mr. Lawrence has found small, long-continued, doses of *hydr. cum cretâ,* frequently of the greatest utility. The same may be said of iodine in those cases in which mercury has disagreed or proved inoperative. Puriform discharge from the *meatus auditorius* is an occasional secondary symptom, yielding to mild mercurials.

Mr. Ormerod's last chapter contains a general summary of the results of his observations upon these two valuable medicines. In treating of *mercury* he properly lays great stress upon the power which its judicious administration exerts in the prevention of secondary symptoms. From his remarks upon the different circumstances which do and do not forbid its use we may extract the following :

"The state of constitution usually denominated strumous is by no means a positive contra-indication to the cautious employment of mercury; if such were the case the frequency of this condition of constitution among the lower orders would often prevent mercurial treatment. In patients whose constitution is weak, and presents the common general signs of scrofula, the employment of mercury is sometimes attended with very great benefit, the patients gaining a degree of flesh and strength under its use which they had not previously enjoyed." P. 281.

The following is the author's general view of the utility of *mercury in primary* disease, his observations on secondary affections we have already quoted.

"The primary indurated, and the rapidly spreading phagedænic ulcers, the indurated cicatrices of old sores, and the glandular swellings accompanied with induration, but with very little surrounding inflammation, are benefitted so much more by mercury than by any other means, that its use is, if not necessary, at least most advisable. The same remedy, though less beneficial in the chronic form of bubo and ulcer, which lasts for months, and sometimes for years, is attended at times with success, and affords more chance of relief than any other means. The cautious employment of mercury in the treatment of a great mass of superficial sores and simple glandular swellings, whether suppurating or not, is attended with no danger, and leads to a good general result by curing a certan number of cases which simple means hardly touch. In acute inflammation of the glands, in acutely inflamed, irritable ulcers, and sloughing phagedæna, the employment of mercury is unattended with benefit, may do harm, and by taking the place of appropriate treatment is to be avoided." P. 233.

In respect of *Iodine* we have the following remarks :

"There does not appear to be at present any very clear proof of the efficacy of iodine in primary disease. This medicine, it is true, has been only partially tried; but the want of any repetition of these trials is a strong argument against the good result derived from its slight use. The greatest advantage derived from iodine in primary disease is seen in the treatment of patients, who from large quantities of mercury, or long standing primary disease, to which secondary symptoms may even be added, are reduced in health. To such, the employment of iodine is certainly beneficial, and aids in restoring them to moderate health. The foul condition of some superficial sores is at times relieved by the employment of iodine, but the local application of the tincture to parts in which considerable swelling and inflammation exist, is sometimes attended with serious injury.

"This small benefit derived from the use of iodine in primary disease is, however, quite compensated for by the advantage gained from its employment in secondary affections. The painful affections and swellings of the bones and periosteum, gonorrhœal rheumatism unaccompanied by much fever, single, foul, phagedænic sores, and large phagedænic sores scattered over the body, are relieved by iodine in a more rapid and a more certain manner than by any other means. Ulcers of the skin, from previous venereal eruptions of any kind, occurring in patients of reduced health, but more especially those following the tubercular and pustular forms, and the conical crusts of rupia, are healed by the internal administration of iodine. The same means have also been found useful after the employment of mercury in the treatment of iritis, as well as in the removal of the protrusion of lymph, which in some cases of iritis perforates the sclerotic at the margin of the cornea. In the papular and scaly eruptions sufficient benefit has followed the employment of iodine to recommend its use, where mercury is objected to, but not enough to render its adoption advisable in preference to that remedy.

" Such are the principal individual symptoms which are relieved by iodine; but the bare enumeration of them conveys but a faint idea of the value of this remedy. These symptoms are some of the most serious and obstinate that occur in syphilitic disease, and which are relieved but little by mercury. These, in fact, are the affections for the cure of which the most varied measures have been introduced at various periods, all of which have enjoyed more or less reputation. The employment of iodine, however, has been attended with greater and more uniform success than any other remedy, except mercury, which has ever been introduced for the treatment of venereal diseases. Those affections which yield least to mercury, and that condition of health which succeeds to long-standing disease and to the employment of very large quantities of mercury, yield to iodine in the most marked and decided manner." P. 290–2.

This is strong testimony, but we believe not stronger than deserved. The opinions of MM. Gibert and Payan upon the subject will be found in the Foreign Periscope of the present Number.

*Hydriodate of Potash* is the preparation of iodine employed at St. Bartholomew's, and it is prescribed in doses of 4 or 5 grains ter.

In concluding our notice of Mr. Ormerod's " Clinical Collections," we may again repeat our opinion that the work does him great credit. It supplies a good and long-wanted account of the practice prevailing in the surgical wards of one of our largest hospitals, and may be advantageously perused by both student and practitioner. Without sympathizing with that hypercriticism which insists upon elegance of medical literary composition, we must still regret that the verbal repetitions and neglect of relative pronouns somewhat mar the pleasure of reading a work otherwise so excellent. This is no doubt due to hasty composition, and may be rectified in another edition.

